# Supplementary material for: The implementation gap: Cross-sector management of heat-related health risks in Western cape, South Africa
Source: PLOS Glob Public Health. 2025 Oct 29;5(10):e0004699. doi: 10.1371/journal.pgph.0004699 (PMC12571260; doi:10.1371/journal.pgph.0004699)
Supplement: S1 File — (DOCX) [file pgph.0004699.s001.docx]

## **Master Interview Guide**

Thank you for taking the time to speak with me today. **Could you please confirm that you agreed to the recording of this interview session?**

*If no, review consent form with participant and stop recording.*

*If yes, continue with recorded interview.*

Thank you. First, I would like to thank you for your support of this research project. The purpose of this interview is to better understand your perspective of how climate risks are managed for health, who works with policies that address climate adaptation and how they are utilized. As described in the consent form and information sheet, what you share with me today in this interview will remain confidential and names will be removed. To start,

1. **Could you please tell me your role and how long have you been in this role?**
2. **What does your role entail?**
   1. **For Academics: where is your work primarily involved? The provincial, national or municipal level?**

Thank you. As you are aware, South Africa, specifically the Western Cape Province, has and will continue to experience increased climate risks such as increasing temperatures and low precipitation leading to heatwaves and wildfires, drought, floods, and sea-level rise.

1. **I am curious to know how important is addressing climate change a part of your work?**

*Probe*

- 1. **Is addressing climate risks a part of your responsibilities/duties? If so, how?**
  2. **What environmental hazards or impacts of climate change do you address in your work?**
  3. **Do you believe you should address climate risks in your work?**

In the field of climate and health, there are climate risks that have a direct impact on health, such as extreme heat, and an indirect impact on health, such as drought. To be more specific, I will now ask you a few questions related to **heat** and **drought** to better understand how decisions are made around these particular climate risks.

1. **Are addressing climate risks such as heat and drought a part of your work responsibilities/duties? Why or why not?**

If yes, ask the following:

- 1. **What were you specifically engaged in?**
  2. **Do you work with other sectors such as the met office or emergency management? If so, what role do they play?**
     1. **Are there stakeholders you feel you should be working with? If so, why aren’t you working with them?**

If no move on to next questions.

1. **Can you tell me about any responses or actions taken at the [governance level] after the 2017/18 Western Cape Drought and the last heatwave?**
   1. **Probe on which heatwave event**
      1. **Were things done differently between heatwave events?**
   2. **Are you aware of any health outcomes that were addressed?**

Interesting, thank you for sharing. There are various policies in South Africa related to climate adaptation and health, respectively.

1. **Of these policies listed here [*show list of related policies- page 5*] which are you familiar with?**
   1. **Which do you work with?**
   2. **Are you responsible for any of these policies?**
2. **Referring back to some of the responses to heat and drought you mentioned earlier, do you feel these are properly addressed in the policies you work with?**

Thank you. I would like to understand how these policies are utilized and implemented, specifically for the health sector.

1. **First, I believe it would be important to understand, what does policy implementation mean to you?**

For the purposes of this interview, I will refer to implementation as ‘the process of coordinating policy or plans, harnessing resources, and decision-making that lead to the execution of actions or the delivery of services’. I will ask you some questions now regarding the policies you indicated you work with.

1. **What can you tell me about the use of [name of policy] in practice? What does this entail?**
2. **What is your/ your department’s particular role in the implementation of policy?**
3. **You are working at the [national/provincial/municipal] level, what is your understanding of the [provincial/ national/ municipal] level role when it comes to implementation?**
4. **Non-health person: Are you aware of health decision makers at the [governance level] that are involved in implementation of climate adaptation policy, such as [*name policy they selected/work with*] and what is their role?**

**Health person: Are you aware of other health decision makers at the [governance level] involved in implementation of climate adaptation policy, such as [*name policy they selected/work with*] and what is their role?**

1. **Do you work with the [*national/subnational*] level when it comes to the implementation of adaptation policy for health? If so, who does what?**

*Probe*

- 1. **Who do you work with/ coordinate on these policies?**
  2. **What kind of information is shared with [*name of sector or governance level named*]?**

1. **Who would you like to be working with, that you aren’t already working with and why?**

*Probe*

- 1. **What kind of information is needed from them?**

1. **Are there other policies that address climate adaptation and health that you are familiar with?**
2. **From your perspective, what are some of the challenges that you have experienced or heard of related to implementation of policies addressing climate adaptation for health?**

*Probes*

- 1. **Are there currently specific issues you encounter related to financing, political will, structures/coordination, or lack of evidence?**
  2. **Do you anticipate any future barriers to implementation?**

1. **Have any activities or policies at the (international/ national/ provincial) level constrained the ability to protect health impacts from climate risks? If yes, how so?**
2. **What factors support the implementation of adaptation actions for health?**

*Probe for further details if necessary*

- 1. **What made those instances possible?**
  2. **Why are some adaptation actions more likely to be taken up or adopted than others?**

1. **What needs to happen for better coordination among responsible actors around implementing adaptation policies for health?**
2. **Are there other stakeholders, even outside government, you recommend I speak with who may provide insight or influence on the implementation of policies addressing climate adaptation as it relates to the health sector?**

I really appreciate you sharing this information.

1. **Before our interview session concludes, is there any other information you would like to share?**

This concludes our interview session. Thank you, again!

*Stop recording and save.*

**List of Policies**

**National**

National Climate Change Response Policy (2011)

National Development Plan (2012)

National Environmental Health Policy (2013)

Climate Change Bill (2018) (2021)

National Climate Change Adaptation Strategy (2019)

National Climate Change and Health Adaptation Policy (2020)

National Heat Health Action Guideline (2020)

**Western Cape Province**

Western Cape Provincial Strategic Plan (2019)

Western Cape Health Strategic Plan (2020)

Western Cape Biennial M&E Report (2020)

Western Cape Climate Change Response Strategy (2021)

**Municipal**

**City of Cape Town**

City of Cape Town Climate Change Strategy (2021)

City of Cape Town Climate Change Action Plan (2021)

City of Cape Town Resilience Strategy (2019)

**West Coast District**

Integrated Development Plan (2020)

West Coast District Climate Change Plan (2019)

**Central Karoo**

Central Karoo Readiness Action Plan (2021)

Climate Change Management Plan (2011)
