# Supplementary material for: The implementation gap: Cross-sector management of heat-related health risks in Western cape, South Africa
Source: PLOS Glob Public Health. 2025 Oct 29;5(10):e0004699. doi: 10.1371/journal.pgph.0004699 (PMC12571260; doi:10.1371/journal.pgph.0004699)
Supplement: Attachment — Submitted filename: Response to Reviewers.docx [file pgph.0004699.s004.docx]

**Response to Reviewers**
The Implementation Gap: Cross-Sector Management of Heat-related Health Risks in Western Cape, South Africa
Journal: PLOS Global Public Health

**Reviewer #1: Comments to the Author** 

Abstract:
The abstract matches the title well in terms of focus and scope.
I looked out for procedural clarity, and methods and process are clearly laid out. E.g.
• Data Source: 31 in-depth interviews.
• Triangulation: Cross-referenced with prior policy document analysis.
• Analytical Framework: Adapted Multiple Governance Framework.
• Levels of Analysis: Provincial and municipal levels, cross-sector stakeholders.
• Outcome: Identification of governance gaps, implementation barriers, and recommendations.
Thus, the procedural description is clear, credible, and appropriate for a governance-focused study.

**Thank you, we appreciate it!**

I would suggest a sentence structure tightening: For example, the last sentence is long and slightly confusing--> "decisionmakers in the Western Cape can manage current and future heat-related health risks that can communities are better equipped..." This is likely a typo or syntax error ("that can" should be removed or rephrased).

**Thank you for this catch! We have improved the final sentence of the abstract and fixed the typo.**

**Introduction:**
For this, I looked out for existing knowledge (which was clearly established). What is unclear is a clear, explicit statement of the research gap. I rather saw an inferred gap (what it seems to be):

Although South Africa has formally recognized heat as a health issue and developed action guidelines, it is unclear how these plans are implemented at the subnational/local level, particularly in terms of cross-sectoral coordination, and whether the top-down approach limits effective governance at those levels.

Clearly state what is missing in existing literature and how the current study addresses that to improve readability

**Thank you for this feedback. We have clarified the research gap in the introduction section (lines 168 -171).**

**Methods:**
I assessed this part using the COREQ (Consolidated Criteria for Reporting Qualitative Research) checklist, and that leaves a lot of gaps which could improve. E.g.

• Almost all Domain 1 (Research Team and Reflexivity) items are missing.
• Little detail on researcher-participant relationship, recording, field notes, or participant validation.
• Data analysis team details (number of coders, how consensus was reached) are absent.
• No mention of data saturation or whether interview guides were piloted or reviewed.

Therefore, while methodologically solid in its sampling and analysis descriptions, the methods section lacks crucial reflexivity and transparency elements common in high-quality qualitative research reporting. Strengthening these areas would improve rigor and replicability.

**I have included additional information on interview guides, re-iterated that the guide was piloted and validated, and provided further information on the analysis of the findings. As this paper is part of a larger research project, these elements were described in more detail in the affiliated thesis document.**

**Results:**
The study aimed to understand how heat-related health risks are managed in the Western Cape, especially at subnational and local levels, and why an implementation gap exists despite national guidelines. The themes are well aligned with this aim and reflect the core focus. The conceptual framing is also appropriate and methodologically sound.

The discussion reiterates the research gap: lack of implementation analysis at subnational levels in South Africa. It reinforces the study’s unique contribution while building on prior studies that framed implementation as overly top-down. This successfully addresses the gap hinted at in the introduction.

**Thank you, this is the gap I clarified in the introduction, per your suggestion. Thanks!**

**Discussion:**
The discussion does not explicitly mention limitations of the study (e.g., generalizability, possible biases from sampling, reliance on self-reported action).
There’s no mention of future research directions or how the findings might be applicable beyond Western Cape.

**Thank you for pointing this out. We have made the limitations a single paragraph in the discussion section, Lines 615 – 627, that has also acknowledged the limited generalizability of the study findings beyond the western cape, but the importance the findings can glean on future research.**

Q1. Is the manuscript technically sound, and do the data support the conclusions?
Yes, the manuscript is technically sound, and the data support the conclusions within the context of a qualitative study. Also, the conclusions are clearly supported by the thematic results. The findings around awareness, system-level coordination, organizational bottlenecks, and individual perceptions all feed logically into the conclusion. The authors are careful not to overgeneralize. Their conclusions focus on the Western Cape context and acknowledge variation across governance levels and sectors. Minor improvements could further strengthen transparency and rigor (COREQ Checklist).
**Thank you very much!**

Q2. Has the statistical analysis been performed appropriately and rigorously?
Since this is a qualitative study, statistical analysis is not applicable. However, I assessed the rigor of the qualitative analysis, which functions as the analytic equivalent in such research. Therefore, I looked at the appropriateness of method used, the framework, coding process, software, transparency and procedural clarity. If COREQ is applied, it will enhance the rigor.
**We have not applied COREQ, and appreciate your acknowledgement of the rigor of this qualitative study.**

Q3. Have the authors made all data underlying the findings in their manuscript fully available?
Only the interview guide is provided. They did state that their data is also provided, although it is not seen here
**Thank you for this, given the ethical approvals and anonymity promised to participants, further data beyond the interview guide cannot be provided as supplementary material.**

Q4. Is the manuscript presented in an intelligible fashion and written in standard English?
The manuscript is presented in an intelligible fashion and written in standard English. However, there are some minor errors. E.g. the last sentence of the abstract earlier mentioned, Lass et al., 1375" seems to be a typographical error so verify and correct the year (it’s likely not 1375), and also minor sentence structuring issues.

**Thank you for catching the typos in the manuscript. It is much appreciated! We have corrected the typos mentioned and caught a few others.**

**Reviewer #2: Comments to the Authors**
This paper offers important insights into the fragmented governance of heat-related health risks in South Africa’s Western Cape province. It shows how limited local engagement, unclear institutional mandates, and weak coordination across sectors have contributed to the poor uptake of the national Heat-Health Action Guidelines. The topic is timely and highly relevant, especially as climate change intensifies heat-related health risks in many low- and middle-income countries. The empirical work is rich and well-grounded in the South African policy context. However, the paper’s contribution could be strengthened by clarifying the research framing, improving the transparency of methods, and making the analysis more accessible to international readers. I offer the following suggestions to improve the paper’s clarity, rigour, and broader relevance.

**Thank you for your review. We have clarified the research gaps and framing and adding information to the methods section. We have responded to the suggestions below in more detail.**

**Major Suggestions**
1. The research question should be stated more clearly. While the paper outlines its focus, it does not explicitly frame a research question. A short and direct research question at the end of the introduction, ideally highlighting the aim to identify barriers and drivers of action, would help orient the reader and clarify the analytical direction.

**Thank you for this suggestion, we have strengthened the final paragraph in the introduction that states the aims of the study to clarify the research question more clearly (Lines 174-176).**

2. The paper explains the sampling and data collection approach, but it does not describe how thematic saturation was assessed. Explaining when and how saturation was reached would improve the credibility of the findings. If saturation was not a central concern, the authors could acknowledge this explicitly or note it as a limitation.

**For the analysis section, we have clarified that themes were reached based on information related to heat as the interviews covered drought as well (Lines 228-229). There was therefore, already a selective set of information on heat to work from. This is further explained in the limitations section in the discussion.**

3. The paper could reflect more critically on the use of the Multiple Governance Framework. The framework is useful for organising the findings, but applying it across all levels of analysis seems to limit the exploration of more complex or cross-cutting themes. For example, the analysis at the organisational level does not fully explore interdepartmental tensions or differences in institutional capacity. The authors could consider whether certain insights might be better presented outside the framework to allow for a fuller discussion.

**In the Methods; Analysis section, it is mentioned that themes were created inductively, and the Framework action domains were applied to glean further understanding. These themes that were found outside of the framework are discussed in the context of the framework action domains, that we find comprehensive. In the example raised around institutional tension, we could not write of this because it was not raised in the interviews in the context of managing heat risks. This is a limitation we have now included more explicitly in the discussion (Lines 615 – 627).**

4. The use of quotes could be clarified. In some places, it is difficult to tell whether a quote reflects a commonly held view or a singular perspective. Where possible, the authors should indicate how many respondents expressed similar views. This would help strengthen the link between the qualitative data and the broader findings.

**Each quote includes a participant number as well as what governance level and sector they represent. In the methods section, we have included that quotes were captured this way for further clarification. For the respondents who held similar views, there is such information provided as part of the analysis regarding the acknowledgement of the heat action plan at the start of the results section (Lines 293 – 295).**

5. The paper would benefit from a visual summary of sectoral roles. Since one of the main findings is that implementation is fragmented across departments, a table or diagram showing which departments are involved, what actions they are taking (or not taking), and how these align with the national guidelines would help make the argument more compelling. It could also help establish the need for cross-sectoral action earlier in the paper. A visual summary of heat-related actions mapped to various departments (even from comparable cities if Western Cape data is limited) would further support this case.

**Thank you for this suggestion, although we agree that this distinction between departments would be helpful, it would identify participants which is against our participant consent form. We allude to the role of different departments in the discussion section and open the study with the importance of working across sectors to address heat. We also feel the table using the adapted Multiple Governance Framework provides another perspective to this distinction across governance levels instead of departments.**

6. The paper could engage more deeply with comparative literature from other heat governance work. India’s experience with national and subnational heat action planning offers valuable points of comparison. For instance, Pillai’s recent work on heat action in India (Pillai, A. V., T. Dalal, I. Kukreti, A. Kassinis, L. V. Zeppetello, E. Tewari & N. K. Dubash. (2025). Is India ready for a warming world? How heat resilience measures are being implemented for 11% of India’s urban population in some of its most at-risk cities. Report. New Delhi.) presents findings that closely align with those in this paper and could be used to draw parallels. The paper would also benefit from referencing literature on heat governance that goes beyond the health sector. Relevant works include:
Meerow, S., & Keith, L. (2021). Planning for Extreme Heat: A National Survey of U.S. Planners. Journal of the American Planning Association, 88(3), 319–334.
Bolitho, A., & Miller, F. (2017). Heat as emergency, heat as chronic stress: Policy and institutional responses to vulnerability to extreme heat. Local Environment, 22(6), 682–698.
Howarth, C., McLoughlin, N., Armstrong, A. et al. (2024). Turning up the heat: Learning from the summer 2022 heatwaves in England to inform UK policy on extreme heat. Grantham Research Institute on Climate Change and the Environment, LSE.

**Thank you for the suggested additional literature. We have included literature on India’s experience with heat action plans, such as Sahi et al 2024 in the discussion. We have expanded the citations to include another source from your recommendations, Pillai et al 2025 (Lines 700 -706). Other literature on health governance beyond the health sector is already mentioned extensively in the introduction and discussion sections of the paper to ensure this paper stays focused on the health sector but acknowledges evidence from related sectors on heat risk management, including from work on cities.**

7. The paper assumes a level of familiarity with South Africa’s institutional context that may limit its accessibility to international readers. A short explanation of the 2020 Heat-Health Action Guidelines, a brief overview of South Africa’s three-tier governance structure, and a sentence or two explaining what Heat Action Plans typically include would help orient readers who are not familiar with the country or the field.

**This information on South Africa’s Heat Action Plan and governance structure is already provided extensively in the Introduction section (Lines 107 – 164). What heat health action guidelines are and what they entail are also included as part of the introduction (Lines 47 to 54).**

**Minor Suggestions**
1. Some quotes appear cut off or lack context, particularly the one on Line 282. Please review all quotations for clarity and completeness.

**The quotes, especially the quote on line 282 (that has since changed) have been slightly amended for clarity.**

2. Line 35 contains a grammatical error (“that can communities are...”) and should be revised. Line 610 should read “system-level.”

**The errors on then line 33 is revised and then line 613 (the title of ‘Coordinating Across sectors (System), has now been corrected to include term “level”. All other sections in discussion have now also been corrected to include ‘level’ as well for consistency.**

3. A table comparing barriers faced by health and non-health departments could help highlight institutional differences in engagement and capacity. This is completely optional, but could help make this more policy oriented.

**Thank you for the suggestion, this indeed would be a helpful table to include. We feel it may highlight a part of this work that is not the main takeaway of the study, so we will therefore not include this in the revised version.**
